# Feasibility and safety of a new endoscopic synthetic sealant nebulizing device over gastric endoscopic submucosal dissections

**DOI:** 10.1007/s00464-021-08480-4

**Published:** 2021-04-13

**Authors:** Ivo Boškoski, Jun Hamanaka, Federico Barbaro, Vincenzo Arena, Pietro Mascagni, Maria Emiliana Caristo, Martina De Siena, Camilla Gallo, Guido Costamagna

**Affiliations:** 1 Digestive Endoscopy Unit, Fondazione Policlinico Universitario Agostino Gemelli IRCCS, Rome, Italy; 2 Centre for Endoscopic Research Therapeutics and Training (CERTT), Catholic University of Rome, Largo A. Gemelli, 8, 00168 Rome, Italy; 3 Department of Gastroenterology, Yokohama Minami Kyosai Hospital, Yokohama, Japan; 4 Area of Pathology, Department of Woman and Child Health and Public Health, Fondazione Policlinico Universitario A. Gemelli IRCCS, Istituto di Anatomia Patologica, Università Cattolica del Sacro Cuore, Rome, Italy; 5 Università Cattolica del Sacro Cuore, Cen.Ri.S, Rome, Italy

**Keywords:** Endoscopic submucosal dissection, Delayed bleedings and perforations prevention, Synthetic sealant nebulizer

## Abstract

**Background:**

Endoscopic Submucosal Dissection (ESD) is the treatment of choice of superficial neoplastic gastrointestinal lesions. Delayed bleedings and perforations are still current clinical concerns. Glubran 2 is a synthetic cyanoacrylate-derived glue nowadays already widely used as an effective tissue adhesive. ENDONEB is a novel device thought for enabling the sealant nebulization over a specific targeted surface during laparotomy, laparoscopy, and thoracotomy. The aim of this single-center preclinical animal trial is to evaluate the feasibility and safety of the same nebulization technique during ESD in the perspective that further clinical studies would demonstrate the efficacy of Glubran 2 in preventing post-ESD adverse events.

**Methods:**

Four live Landrace pigs were enrolled. Two approximately 30-mm-wide gastric ESDs were performed in each pig (experimental ESD and control ESD). About 0.5 mL of Glubran 2 was nebulized on the experimental ESDs. Subjective perception of the feasibility of the Glubran 2 nebulization was reported. Pigs were clinically monitored at follow-up and upper GI endoscopy was performed at 24 and 48 hours, when animals were euthanized to perform a macroscopic and histological analysis of the specimens.

**Results:**

No peri-procedural adverse events were reported. Glubran 2 nebulization over experimental ESDs showed to be technically easy and time-effective. Clinical and endoscopic animal monitoring was negative at follow-up. At 24 hours, the Glubran 2 film was clearly visible on the eschar of the ESDs and signs of initial hydrolysis were discernable at 48 hours. No signs of peritoneal reaction were observed at the macroscopic examination. Equal transmural inflammation was described at the histological examination of both types of ESDs.

**Conclusions:**

Safety and feasibility profiles of Glubran 2 nebulizing ENDONEB device over ESD surfaces were excellent. Further evidences and human trials are needed to investigate its effectiveness in ESDs’ eschars sealing and, thus, in delayed micro-perforations and bleedings prevention and treatment.

**Supplementary Information:**

The online version contains supplementary material available at 10.1007/s00464-021-08480-4.

Endoscopic Submucosal Dissection (ESD), first described in Japan about two decades ago [1], is an advanced endoscopic procedure for en bloc resection of superficial neoplastic gastrointestinal (GI) lesions. ESD was initially applied to remove early gastric cancers (EGC) with no or low probability of lymph node metastasis and nowadays is widely used also for esophageal and colonic lesions. According to the guidelines of the Japanese Gastroenterology and Endoscopy Society, endoscopic treatment of EGC is indicated to remove intramucosal (cT1a) differentiated carcinomas that are less than 2 cm in diameter with no findings of ulceration [2]. Both the European and American Societies of Gastrointestinal Endoscopy recommend ESD over Endoscopic Mucosal Resection (EMR) for most EGC lesions, given the higher rate of complete en bloc resections. However, gastric ESD is technically complex to perform and it is related to significantly longer operation time and higher rate of intraoperative and delayed bleedings and perforations compared to EMR [3, 4]. Indeed, despite the use of Proton Pump Inhibitors (PPIs) and the prophylactic endoscopic clipping and coagulation of visible vessels, delayed bleedings may still occur after ESD (up to 9%, 2.7% and 1.7% for gastric ESDs [5–7], colonic ESDs [8] and Barrett’s ESDs [9, 10], respectively).

Several strategies have been proposed to manage ESD-related post-operative complications [5, 11–13]. Tissue sealants and biological adhesives glues, such as PuraStat (PuraStat; 3D Matrix Ltd, France), a self-assembling peptide [14], EndoClot, a polysaccharide hemostatic system (EndoClot® PHS) [15], and Hemospray® (Cook Medical), a mechanical hemostatic agent [16], are nowadays routinely used either surgically or endoscopically to treat gastrointestinal perforations and bleedings [17] and they have been demonstrated to be effective in preventing delayed gastrointestinal re-bleedings [18]. Similarly, Glubran 2, a biocompatible acrylic sealant constituted by the two *N*-butyl-2-cyanoAcrylate and methacryloxy-sulfolane monomers (manufactured by GEM S.r.l., Viareggio Italy), is currently vastly used thanks to its hemostatic properties, fast action and adhesive strength [19]; this sealant polymerizes in 1–2 seconds to 1 minute when in contact with tissues, generating a tight transparent film that is permeable to oxygen but not to liquids [20]. Its efficacy has been tested mainly in preclinical animal studies: Glubran 2 showed to be useful for the neuroradiological endovascular treatment of fistulas and AVMs [21] and management of possible complications arising during the embolization of aneurysms [22]; it also demonstrated to be successful as an innovative strategy for mesh fixation following surgical abdominal hernia repair [23–26]. Glubran efficacy has not only been demonstrated on animal tissues, but also directly on humans: the first preliminary experiences were collected on a pediatric sample subjected to laparoscopic endosurgery [20]. The endoscopic application of Glubran 2 firstly occurred as a successful rescue treatment for external pancreatic fistulas resistant to conventional endoscopic drainage therapy [27].

Mainly thanks to its physical properties and the clinical implications of its use, this synthetic sealant could be thought not only as a therapeutic mean but also as an effective prophylactic strategy to prevent or even seal delayed bleedings and micro-perforations of ESD surfaces, also considering the fact that a prospective study focused on post-ESD delayed bleedings prevention conducted on humans demonstrated the effectiveness of a pure monomeric butyl α-cyanoacrylate adhesive very similar to Glubran 2 in its chemical composition and properties (0% vs. 4.88% in the control group; *p* = 0.035) [28]. For this purpose, a Glubran 2 nebulizing device called ENDONEB has been developed and patented; it is CE mark pending. The ENDONEB device (Fig. 1) consists of disposable applicator, composed by a 2 m catheter, which is connected to a handpiece where a syringe containing Glubran 2 is positioned, and to a propellant cylinder containing a non-toxic and non-flammable gas (HFC134/a 1,1,1,2 tetrafluoroethane). The catheter is arranged inside a sheath and contains two small tubes, one for the passage of the gas and one for the passage of Glubran 2, which both flow into a single diffuser tip.

**Fig. 1:**
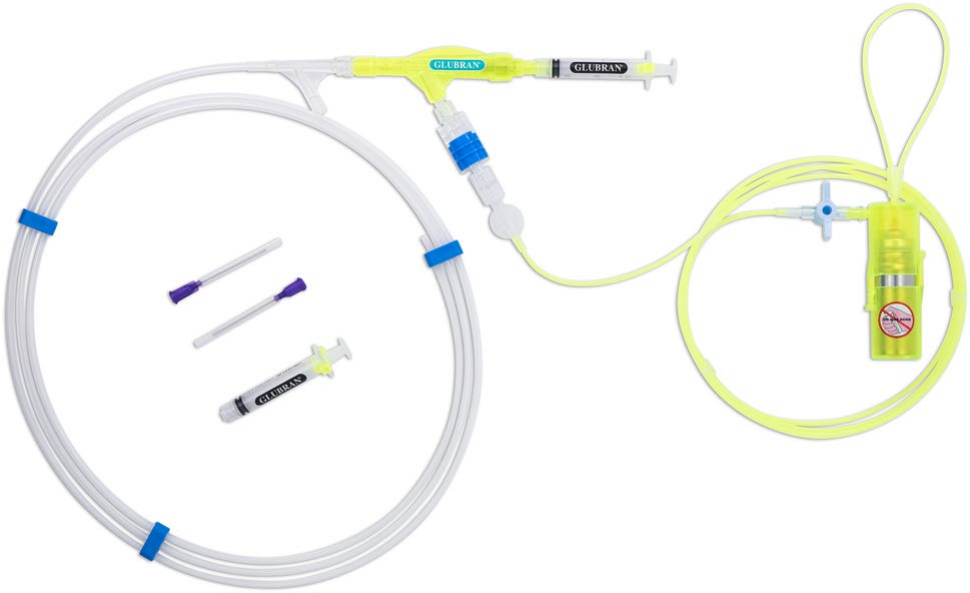
The ENDONEB nebulizing device

To date, there is no scientific evidence available regarding the application of Glubran 2 on the surface of the ESD eschar, nor even objective data reporting the feasibility of the ENDONEB Glubran 2 nebulizator. The main aim of our preclinical animal trial is, thus, to evaluate feasibility and safety of Glubran 2 endoscopic nebulizing over gastric ESD surfaces with the ENDONEB device. Feasibility is intended as not excessive extension of the procedural time, uncomplicated way of using the ENDONEB device and easy addressing of the nebulized sealant jet; safety is intended as low intra-procedural and post-procedural adverse events incidence rate. The endoscopic appearance of the ESD at follow-up and necropsy and histological appearance of the extracted specimens are considered as secondary outcomes of our study.

## Materials and methods

### Study design

A single-center prospective preclinical feasibility and safety trial was performed in live pigs. All animals were managed according to the Italian laws for animal use and care and according to the directives of the European Community Council (2010/63/EU). The study was approved by the local ethics committee (1F295.86) and by the Italian Ministry of Health (464/2020-PR). In order to study the Glubran 2 nebulized application feasibility and safety, two equally wide ESDs were performed in the gastric body of each enrolled pig: one of these two ESDs was performed according to the traditional technique that does not require the application of any sealants on its surface (the “control ESD”), while Glubran 2 was applied on the eschar of the other ESD through the ENDONEB nebulizer (the “experimental ESD”). Any peri-procedural or delayed adverse event has been reported and the endoscopist’s perception of the feasibility of the experimental technique has been noted. The clinical, endoscopic and histopathological aspect of the ESDs was described.

### Animals and endoscopic procedure

Four Landrace pigs (100% females, weight range 30–35 kg, age range 3–4 months) were utilized in this study. Each pig fasted from the day before the procedure with unlimited access to water. Tiletamine/Zolazepam (7.5 mg/kg, Zoletil 100, Virbac, France) and Azaperone (2 mg/kg, Stresnil, Janssen‐Cilag, Belgium) were administered intramuscularly as premedication 10 min before the procedure. Induction of anesthesia was achieved by using intravenous Propofol (3 mg/kg) combined with Rocuronium (0.8 mg/kg). After endotracheal intubation of the animals in the supine position, anesthesia was maintained with 2% Isoflurane.

An experienced endoscopist (J.H.) performed 2 ESDs (an “experimental ESD” and a “control ESD”) approximately 30 mm in diameter in the gastric body of each animal. A 10 mm snare was mainly used to standardize the size of each ESD: once it was opened at the site where the ESD was meant to be performed, the width equal to three times the maximum opening of the snare itself was measured by eye and the delineation of the desired resection margins was, thus, performed using a DualKnife™ (Olympus, Tokyo, Japan). After the delineation of the desired margins, each ESD was performed following the reported main procedural steps: (1) mucosal lifting via submucosal injection (23 G needle, Interject™ Contrast, Boston Scientific, MA, USA) of a physiological saline (NaCl 0.9%) solution dyed with drops of methylene blue, (2) precut mucosal incision, (3) submucosal dissection alternating the DualKnife™ and an Insulated Tip (IT) Knife (KD-612U; Olympus, Tokyo, Japan) and (4) extra submucosal injections if necessary, (5) careful inspection of the gastric resection bed and (6) extracted specimens macroscopic examination.

### Glubran 2 application

To complete the procedure on each “experimental ESD”, the ENDONEB device was connected to the gastroscope. To make proper use of this device, the endoscopic catheter protective sheath has to be removed and the catheter itself has to be inserted in the operative channel of the gastroscope till 2 to 3 cm from the tip of the same gastroscope, avoiding direct contact with the gastric mucosa. To nebulize the Glubran 2, the gas cylinder positioned at the ENDONEB handpiece needs to be opened and the piston of the syringe containing the sealant has to be pressed. In this way, the propulsive activity of the gas is exploited to precisely target the nebulized Glubran 2 over the desired surface. In our specific case, an amount of about 0.5 mL of Glubran 2 was applied on the eschar of each “experimental ESD” in order to create a proper sealant film. (Video 1) The experienced endoscopist was asked to report in detail his reliable subjective perception of the feasibility of the Glubran 2 nebulization over the “experimental ESDs” compared to the traditional technique referring to the procedural time, to the ease of use of the ENDONEB device and to the precision of the nebulized sealant jet.

### Post-operative management and follow-up

During the post-operative period, the animals had food and water access ad libitum. No oral PPIs nor medications were given to the pigs after ESD and during the follow-up time. Pigs were directly observed and clinically monitored for possible macroscopical bleedings or any other adverse event. The ESDs follow-up was conducted by performing upper GI endoscopy at Post-Operative Day (POD) 1 at 24 hours and POD 2 at 48 hours. At POD 2, all pigs were euthanized with an intravenous injection of a lethal dose of Embutramide/Mebenzonium Iodide/Tetracaine Hydrochloride (Tanax®, T-61). Each animal’s stomach was subsequently removed, and necropsy and histological analysis of the obtained lesions was carried out.

## Results

**Fig. 2:**
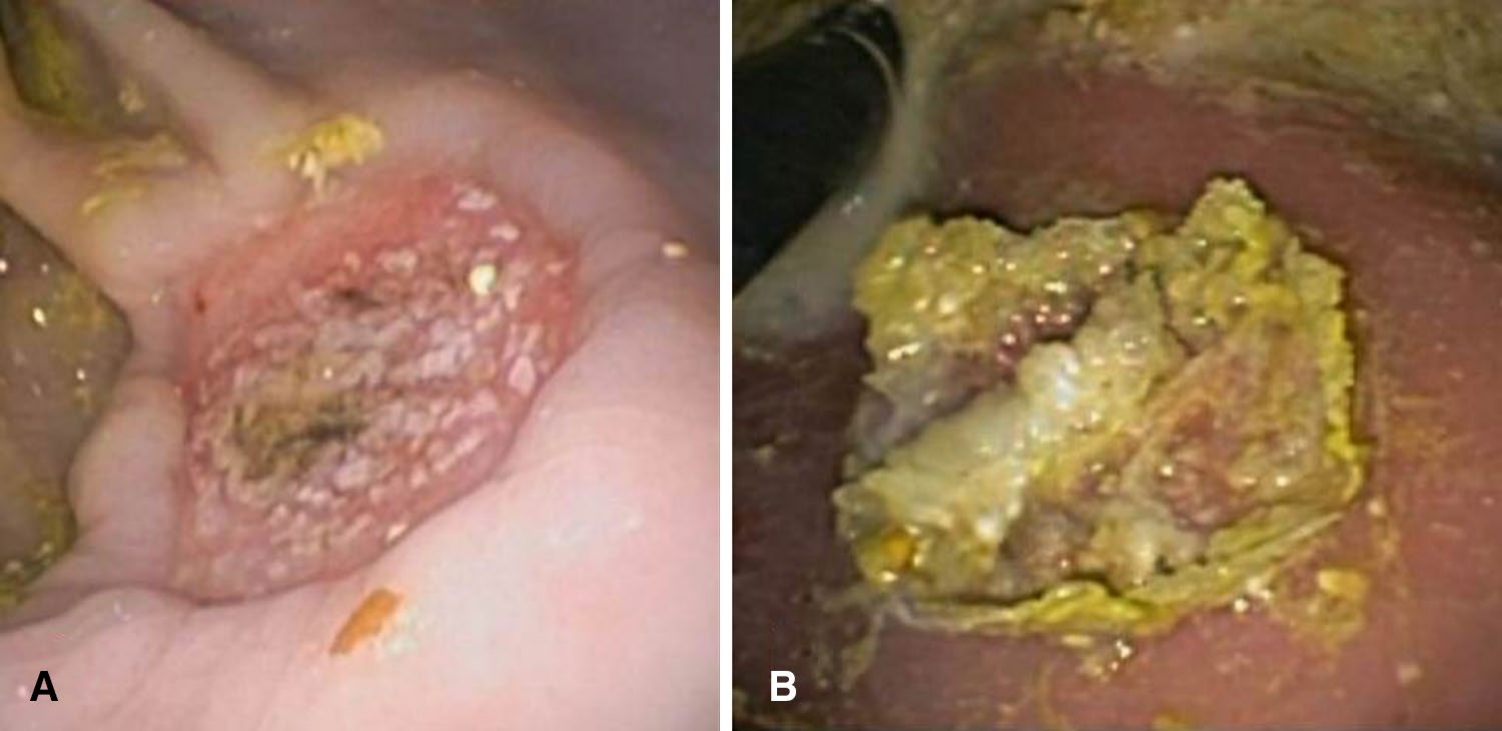
Post-Operative Day 1: Glubran 2 film clearly visible at the upper GI endoscopy on the eschar of the experimental ESD (**a**) and endoscopic aspect of the control ESD (**b**)

**Fig. 3:**
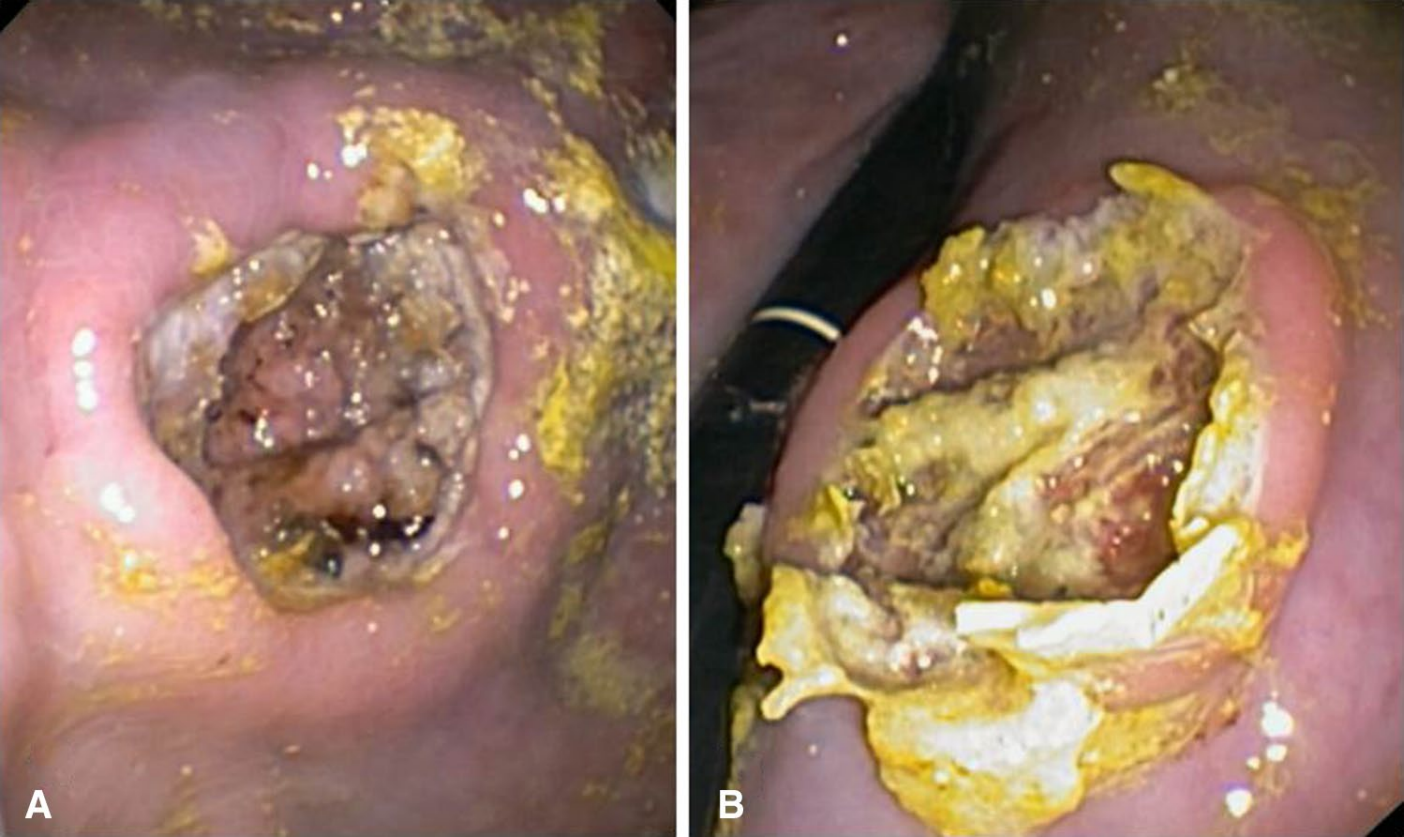
Post-Operative Day 2: normal endoscopic aspect of the control ESD (**a**); initial hydrolysis of the Glubran 2 film on the eschar of the experimental ESD (**b**)

**Fig. 4:**
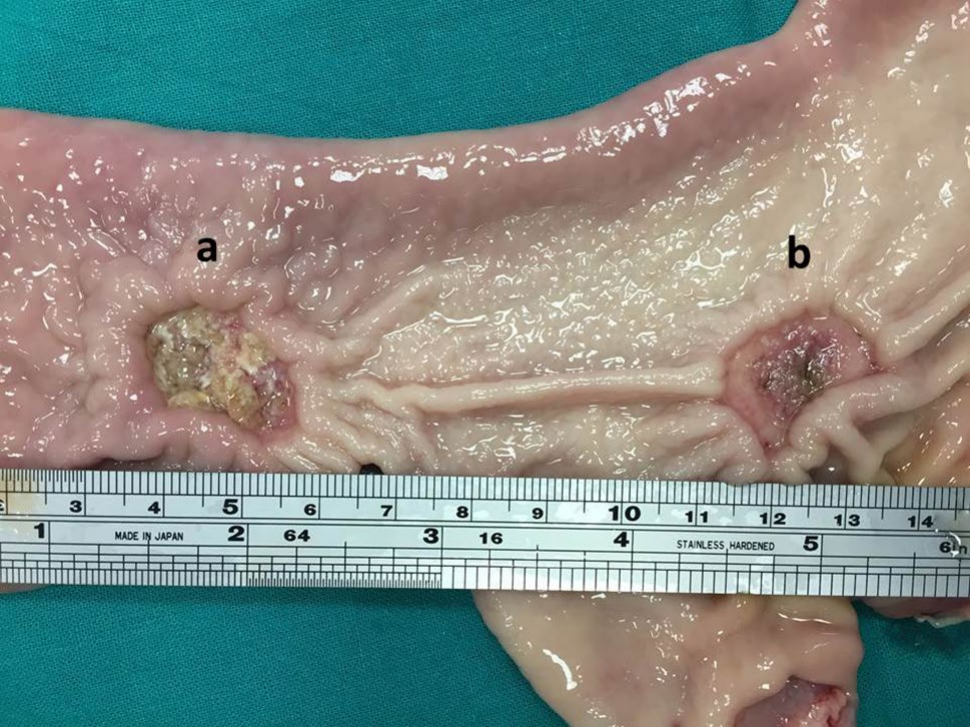
Macroscopic aspect of both types of ESD: experimental ESD (**a**) and control ESD (**b**)

**Fig. 5:**
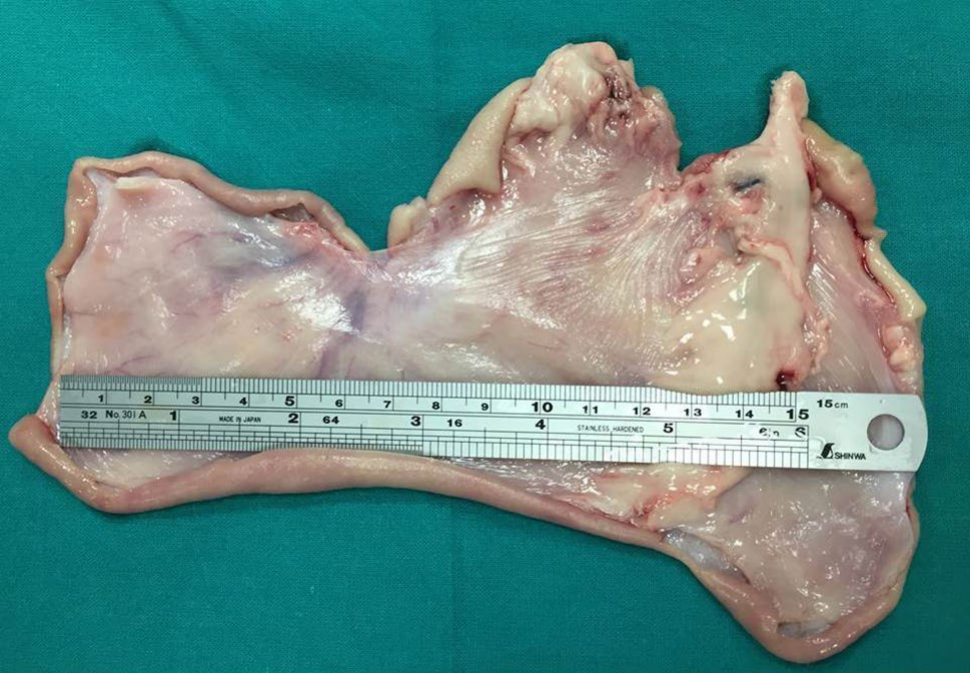
Macroscopic aspect of the harvested specimens: no sign of peritoneal reaction on the serosa side of both types of ESD

**Fig. 6:**
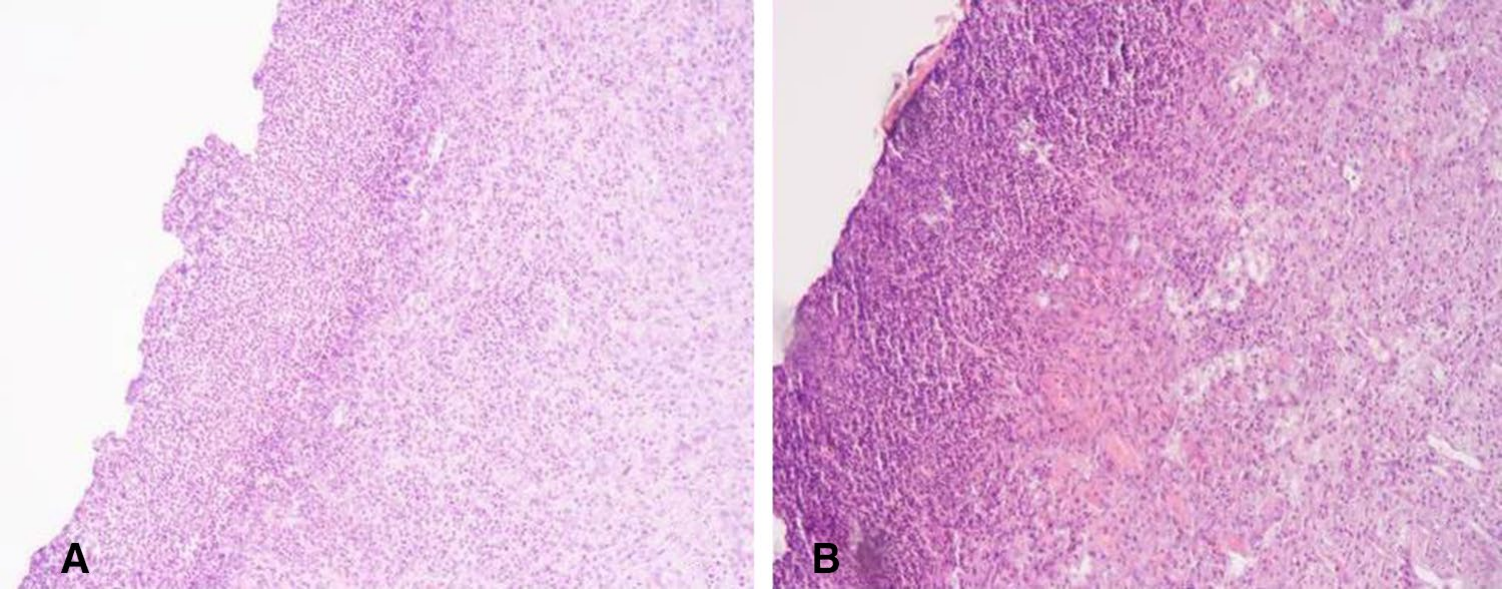
Histological finding of equal transmural inflammation and ulcerations in experimental ESD (**a**) and control ESD (**b**)

Considering both types of ESD performed with or without the application of the synthetic sealant, no bleedings nor other adverse events were reported during the peri-procedural time. Mean ESD time was 45 min (range 38–55). The endoscopist reported that, compared to the traditional technique, nebulizing the Glubran 2 over the ESD surface does not excessively influence the procedural time, and, thus, does not expose the animal models to increased procedural-related risks. The ENDONEB device showed to be uncomplicated to use and the nebulized sealant jet appeared to be easily addressable on the specific desired surface. All four animals enrolled in the study survived during the post-operative period and their clinical monitoring and direct observation were all negative. None of the eight ESDs performed showed signs of bleeding at upper GI endoscopy follow-up, but visually evident differences were highlighted comparing the endoscopic aspect of the experimental ESDs to the one of the control ESDs. At POD 1, in particular, the Glubran 2 film was clearly visible at the upper GI endoscopy on the eschar of the experimental ESDs compared to the control ESDs (Fig. 2a, b) and at POD 2 an initial process of hydrolytic breakdown of the Glubran 2 film was discernible on the eschar of the experimental ESDs. (Fig. 3a, b) Traces of the crystalline-like sealant and of its ongoing gradual hydrolysis were confirmed at necroscopy of the harvested tissue specimens (Fig. 4) and no signs of peritoneal reaction on the serosa side of both types of ESD were discerneable (Fig. 5). The macroscopic naked eye and endoscopic images observed correspond, according to the scientific literature, to the initial stages of hydrolytic degradation of the Glubran 2 film into water-soluble remains [19, 22], which is a process that ends in a time ranging from 30–40 days to 6 months depending on the amount of sealant deposited and on the thickness of the sealant film [20, 22, 25]. Transmural inflammation and ulcerations were described at the histological examination of both types of ESDs, with no evidence of specific differences between the two (Fig. 6). Glubran 2 is, in fact, not associated with foreign body reactions [20], and it does not compromise the healing process [24].

## Discussion

ESD technique has undoubtedly revolutionized the treatment of GI superficial neoplastic lesions. Compared to EMR, ESD is technically more difficult and, despite the traditional prophylactic measures such as PPI use and visible vessels coagulation or clipping, it is still associated with an increased risk of delayed bleeding. Tissue sealants and biological adhesive glues directly applied on GI lesions represent promising methods of delayed bleedings prevention and treatment, but clinical evidence is still not strong enough.

The nebulized Glubran 2 consists of biocompatible acrylic sealant already widely applied as a therapeutic strategy in surgery, interventional radiology and endoscopy. It is composed of two monomers: *N*-butyl-2-cyanoacrylate and methacryloxy-sulfolane. The addition of the Methacryloxy-Sulfolane makes the compound more stable and even more biocompatible if compared to the initially used pure butyl α-cyanoacrylate monomer, inducing the releases of non-toxic components during the biodegradation process [28]. Moreover, the polymerization reaction of Glubran 2 in contact with organic tissues leads to a maximal local temperature of 45 °C [28], which appears to be much lower than the local temperature reached with the exothermic chemical reaction induced by the single butyl α-cyanoacrylate monomer, that can rise up to 80 °C [29].

As observed in our study follow-up, the persistence of the sealant film over the ESDs eschars was clearly evident at the endoscopic examination at POD 1 (24 hours) and also still at both the necroscopic and endoscopic examination at POD 2 (48 hours) (Figs. 3a, b and 4). This evidence could have a very important clinical significance, since the onset of delayed ESD-related bleedings in humans rates up to 1.6% in the first 48 hours and gradually decreases proportionally to the passage of time [30]. The observed evidences could therefore provocatively open the way to the application of this sealant as a standard method of ESD-related complications prevention, such as mainly delayed bleedings and micro-perforations. However, further clinical evidence deriving from human trials is needed to prove the hypothesis.

The results of our study, although preliminary, confirm the feasibility and safety of using this sealant by means of an effective nebulizing device. The ENDONEB was, in fact, shown to be safe and extremely easily exploitable on ESDs surfaces. The number of animals and ESDs performed was chosen in order to reduce at minimum animal suffering and it was sufficient for obtaining preliminary data. Using a larger porcine sample or even replicating this study on ESDs performed in humans could increase the strength of the evidence reported.

In conclusion, even if this study could not demonstrate the efficacy of the Glubran 2 in preventing ESD-related delayed bleedings and micro-perforations, safety and feasibility profiles of this synthetic sealant ENDONEB nebulizer device proved to be excellent over ESDs surfaces.

## Supplementary Information

Below is the link to the electronic supplementary material.

Video 1. Nebulized Glubran 2 applied on the eschar of the experimental ESD. Supplementary file1 (MP4 25410 kb)
